# Testing the capacity of humanized immune system mice to induce a protective antibody response against the Lyme disease pathogen

**DOI:** 10.1128/spectrum.03803-25

**Published:** 2026-04-07

**Authors:** Natanel Neumann, Moriah L. Jacobson, Michael A. Brehm, Keith A. Daniels, Jamie Kady, Carey L. O'Donnel, Yuliya Rogovska, Sichao Wang, Artem S. Rogovskyy

**Affiliations:** 1 Department of Pathobiology and Diagnostic Investigation, College of Veterinary Medicine, Michigan State University 573378, East Lansing, Michigan, USA; 2 Taconic Biosciences, Rensselaer, New York, USA; 3 Program in Molecular Medicine, The University of Massachusetts Chan Medical School 378174, Worcester, Massachusetts, USA; 4 Center for Statistical Training and Consulting, Michigan State University 336694, East Lansing, Michigan, USA; University of Florida, Gainesville, Florida, USA

**Keywords:** Lyme borreliosis, animal models, humanized mice, antibody response, vaccine candidates

## Abstract

*Mus musculus* has been a key animal model whereby important discoveries on bacterial pathogenesis of the Lyme disease (LD) pathogen, *Borreliella burgdorferi* (*Bb*), were made. In the United States alone, LD affects the lives of about half a million new patients each year, making it the most prevalent tick-borne illness. To date, however, there is no LD vaccine for humans on the market, and therefore, much research effort has focused on identifying protective *Bb* vaccine candidates. Because murine and human immune responses to *Bb* significantly differ, we evaluated humanized mice to improve the translatability of preclinical Lyme vaccine studies. Specifically, we assessed two humanized mouse models, UCB-HSC NSG and huNOG-EXL SA, for their capacity to induce protective anti-*Bb* antibodies. For that, we utilized an antibody-susceptible *Bb* (∆VlsE), which is effectively cleared by acquired anti-*Bb* antibodies of immunocompetent mice; and outer surface protein A (OspA), a surface *Bb* antigen that proved efficacious not only as part of licensed canine vaccines but also as part of a human LD vaccine that was transiently available to the public in the past. The data demonstrated that the tested models could not clear the ∆VlsE infection. Likewise, OspA-vaccinated humanized mouse immune system failed to prevent wild-type challenge. Given that no better alternative preclinical model has been developed, we hope that the inherent translatability limitations of standard LD mouse models will continue to be favorably accepted. Although our results do not encourage the use of the tested humanized models for preclinical LD vaccine efficacy testing, their utility remains valuable for mechanistic studies.

**IMPORTANCE:**

In this study, we assessed the ability of mice with humanized immune systems to develop protective antibodies against the bacterial agent of Lyme disease (LD), *Borreliella burgdorferi*. In contrast to an intact mouse immune system of classical LD models, the humanized immune system could not prevent vaccinated mice from being infected with LD spirochetes. The findings demonstrate that two tested humanized models cannot be used for preclinically testing LD vaccine candidates. Given the results, we hope that the animal-to-human translatability limitations of existing LD mouse models will be more favorably taken as there is no better alternative preclinical model developed.

## INTRODUCTION

Lyme disease (LD), also known as Lyme borreliosis, is caused by spirochetes of *Borreliella burgdorferi* (*Bb*) sensu lato complex, representing the most significant tick-borne illness in North America (1). Climate change is thought to have been implicated in the spread of the spirochetal vector, *Ixodes* spp. ticks, increasing incidence rates of LD (2, 3). LD is a multisystemic disease of debilitating nature partly due to the capacity of Lyme spirochetes to establish a lifelong persistence (4–9) in spite of strong anti-*Bb* antibody responses (10–13). Early non-specific (flu-like) symptoms are often missed when the pathognomonic erythema migrans lesion has not developed. Chronic LD stage may manifest itself as arthritis, carditis, encephalitis, meningitis, and/or musculoskeletal pain (14). The antibiotic treatment of late LD could be unrewarding. Paradoxically, to date, in the United States (U.S.), there is no LD vaccine available for humans, yet veterinary (canine) vaccines have been on the market (15–20).

In humans or mice, *Bb* spirochetes have the ability to survive robust anti-*Bb* antibody responses by utilizing a highly effective antigenic variation system encoded by the variable major protein (VMP)-like sequence (*vls*) locus (21). The *vls* locus, which is well studied for *Bb* strain B31, is housed by linear plasmid lp28-1 (21). Recombination within the locus between the *vlsE* gene and 15 non-expressible cassettes produces variations of *vlsE* sequence, which are sufficient for productive antigenic changes of surface-localized lipoprotein, VlsE (22). Genetically deleting the *vlsE* locus disables *Bb* spirochetes (∆VlsE) to evade anti-*Bb* antibodies. When challenged with the ∆VlsE mutant, immunocompetent mice clear its transient infection within 14–21 days, whereas immunodeficient mice become persistently infected (23–31).

To date, there are two well-established animal models to study LD—the non-human primate (NHP) and the mouse. The NHP model has been used in the LD research field (32–35) primarily because this model mimics human LD manifestations (e.g., Lyme neuroborreliosis) more closely than mice (33, 35–40). However, due to its numerous caveats (e.g., ethical concerns, reproducibility issues, reagents unavailability, costs, and limited facilities), the utility of the NHP model is significantly restricted. Among laboratory mouse strains, C3H mice are likely to have been the most common mouse model used in the LD field. Similar to LD patients, upon *Bb* infection, C3H mice develop strong anti-*Bb* antibody responses and yet consistently fail to clear wild-type infection, developing *Bb*-induced arthritis and carditis (41–44). However, LD mouse models also have their own sets of limitations. For example, humans and mice differ in their baseline immune architecture, such as having different immunoglobulin subclass repertoires as well as differing proportions of neutrophils and lymphocytes present in the peripheral blood (45). Clinically, humans commonly develop erythema migrans, whereas mice do not (46). Furthermore, human LD-induced lesions are predominantly infiltrated with lymphocytes, whereas the respective mouse lesions are initially enriched with innate immune cells (46). Recognizing that both NHPs and mice offer distinct advantages and limitations with respect to human translatability and feasibility, we set out to see whether a humanized immune system in mice could provide an intermediate platform to help bridge the differences in LD vaccine efficacy evaluation.

Mice with a human immune system (humanized mice hereafter) have been used as a preclinical bridge for various biomedical research applications (47). Humanized mice are generated by engrafting immunodeficient mice with human peripheral blood mononuclear cells (PBMCs), hematopoietic stem cells (HSCs), or human fetal tissues (thymus or liver) (47, 48). Humanized mice have been extensively utilized to study human innate and adaptive immune responses against different microbial infections (49–52). In addition to viral and fungal pathogens, numerous studies successfully applied humanized models to characterize human immune responses against various bacterial pathogens (e.g., *Salmonella*, *Mycobacterium tuberculosis*, *Staphylococcus aureus*, *Escherichia coli*, and *Neisseria gonorrhoeae*) (53–65). However, the use of humanized mouse models can lead to unanticipated results. Prior reports show that humanized mouse platforms can yield phenotypes of host-pathogen interactions that deviate from inbred mouse results (62, 66). Furthermore, despite similar host backgrounds, different humanized mouse models display different pathology and mortality when evaluating drug toxicity (67, 68). Importantly, humanized mice have been used as an effective evaluation system for some human vaccines (69). For example, several studies tested antigens of human pathogens showing production of protective antigen-specific antibodies (70–76). Promisingly, an investigation using humanized mice infected with a tick-borne relapsing fever (RF) spirochete, *Borrelia hermsii*, also demonstrated development of *B. hermsii*-specific antibodies responsible for spirochetemic relapses, the hallmark of human RF disease (77). Encouraged by the results of previous investigations, in the present study, we set out to test whether humanized mice have the capacity to develop a protective antibody response against *Bb*.

In this study, we have utilized two HSC-engrafted humanized mouse models based on immunodeficient NOD-derived backgrounds (UCB-HSC NSG and huNOG-EXL SA). Both models carry the *Prkdc^scid^* mutation, preventing murine B- and T-cell development and mutations in the IL2 receptor common gamma chain (*Il2rg*), causing deficiencies in NK cell development and impaired murine cytokine signaling (78). huNOG-EXL SA mice are based on the NOG-EXL background, whose transgenic expression of human GM-CSF and IL-3 cytokines supports human myeloid-cell differentiation and improves overall engraftment success and human immune reconstitution compared to the base NOG or NSG mouse models (79). Taken together, our findings indicate that the humanized mouse platforms tested in this study did not generate any protective anti-*Bb* antibody responses and therefore are not yet ready to replace classical mouse models for preclinical LD vaccine efficacy testing.

## MATERIALS AND METHODS

### Bacterial strains

A wild-type strain of *Borreliella burgdorferi* B31-A3 (B31) and its parental previously generated mutant B31-A3∆*vls* (∆VlsE), which were generous gifts from George Chaconas and Troy Bankhead (28), were cultivated in Barbour-Stonner-Kelly medium supplemented with 6% rabbit serum (Pel-Freeze Biologicals, AR, USA) and antibiotic cocktail (0.02 mg mL^−1^ phosphomycin, 0.05 mg mL^−1^ rifampin, and 2.5 mg mL^−1^ amphotericin B) at 35°C under 2.5% CO_2_. The medium with the serum and antibiotic cocktail is referred to here as BSK-II medium.

### Humanized mice

Female NOD.Cg-*Prkdc^scid^ Il2rg^tm1Wjl^*/SzJ (NSG mice, JAX # 005557, the Jackson Laboratory, ME, USA) mice of 3–4 weeks of age were conditioned with a dose of 100 cGy and then intravenously injected with ~1 × 10^5^ human CD34^+^ hematopoietic stem cells (HSCs) derived from umbilical cord blood (UCB) (80). UCB was obtained in accordance with the Committee for the Protection of Human Subjects in Research guidelines of the University of Massachusetts Chan Medical School. UCB was provided by the medical staff of the University of Massachusetts Memorial Umbilical Cord Blood Donation Program. Engraftment of HSC was confirmed by flow cytometric analysis as described (80). A total of three different UCB donors for UCB-HSC NSG mice (HuCB 1946.1, HuCB 1957.2, and HuCB 1957.3) were utilized. NOD.Cg-*Prkdc^scid^* Il2*rg^tm1Sug^*Tg (SV40/HTLV-IL3,CSF2)10-7Jic/JicTac (huNOG-EXL SA) mice generated by using four donors (007, 55, 56, and 127) were graciously provided by Taconic Biosciences (NY, USA). The animal ages and dates of engraftment are provided in Tables S1 and S2.

### Mouse infection with B31 and ∆VlsE

**Fig 1:**
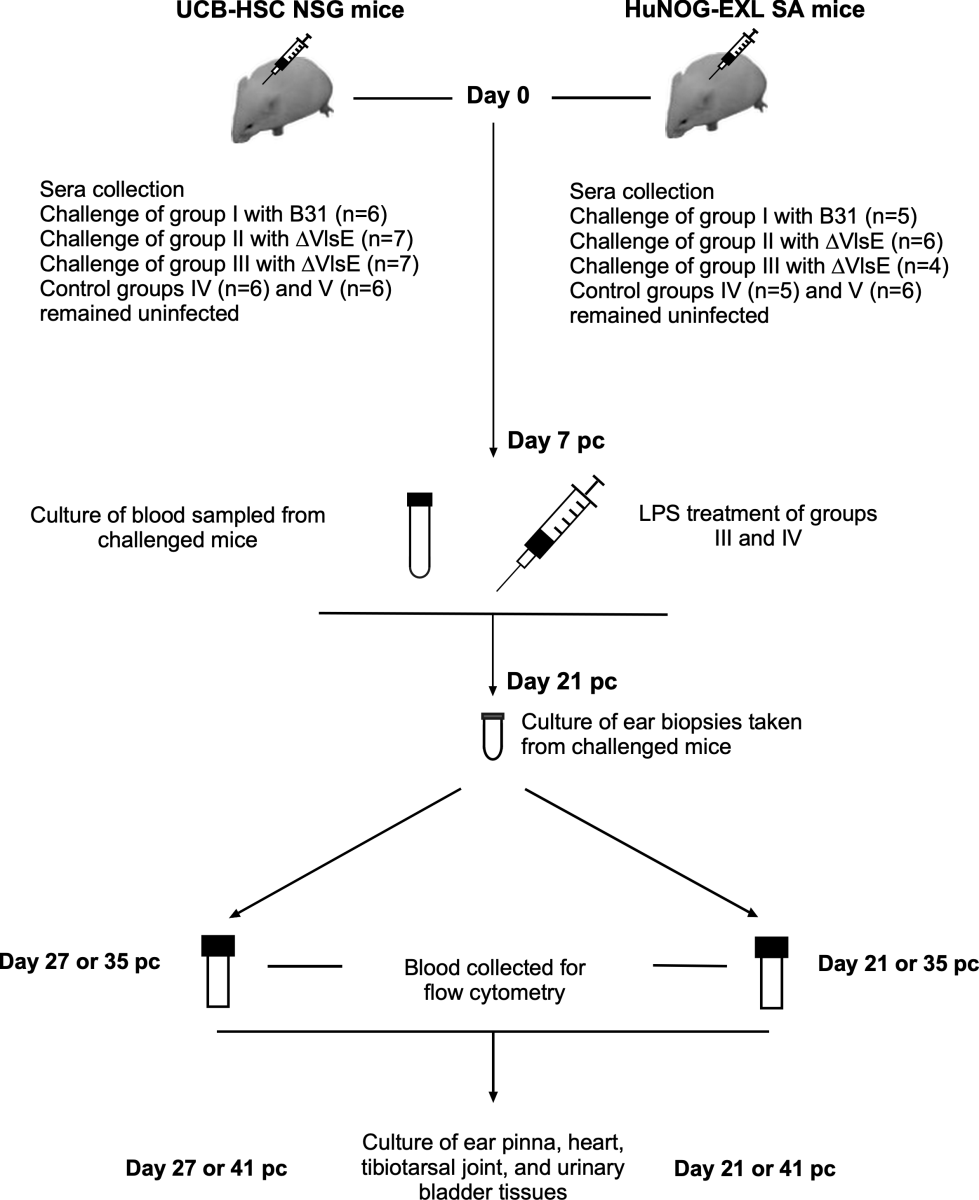
The experimental design of the infectivity study evaluating the immune responsiveness of humanized mice to *B. burgdorferi*. Two humanized mouse models, UCB-HSC NSG and HuNOG-EXL SA, were split into five groups (the group sizes are indicated in the figure). Group I mice were subcutaneously (s.c.) challenged with *B. burgdorferi* B31-A3 (B31), whereas animals of groups II and III were s.c. challenged with B31-A3Δ*vls* (ΔVlsE). Groups IV and V served as uninfected controls. At day 7 postchallenge (pc), blood was collected from each challenged mouse for culture, and groups III and IV mice also received lipopolysaccharide (LPS). At day 21 pc, ear pinna biopsies were sampled from each challenged mouse of groups I, II, and III for culture. Blood for flow cytometry analysis was collected from UCB-HSC NSG mice at day 27 or 35 pc and from huNOG-EXL SA mice at day 21 or 35 pc. The dates of sacrifice varied because of the health status of donor-specific mouse subgroups. Upon sacrifice, ear pinnae, hearts, tibiotarsal joints, and urinary bladders were collected for culture to assess the outcome of each challenge.

Females of each humanized mouse model, UCB-HSC NSG and huNOG-EXL SA, were split into five groups (Fig. 1). At day 0, each animal of the first three groups was subcutaneously (s.c.) challenged with a 100 μL inoculum (~1 × 10^5^ spirochetes per mouse) with B31 (group I) or ∆VlsE (groups II and III) in the shoulder area. At day 7 postchallenge (pc), groups III and IV mice were injected with lipopolysaccharide (LPS; LPS-B5 Ultrapure; InvivoGen, CA, USA) through retro-orbital injection at a dose of 1 µg per 1 g of body weight (~58–130 µL per mouse). Group IV animals of both UCB-HSC NSG and huNOG-EXL SA mice were never exposed to *Bb* as they served as LPS-treated animal controls. Group V animals remained untreated and uninfected, serving as uninfected control groups. At day 7 pc, 30 μL of blood collected from each challenged mouse via cheek bleed was cultured in 3 mL of BSK-II medium to monitor borrelial infections. Blood cultures were examined via dark-field microscopy (DFM) weekly for up to 2 weeks for the presence of viable spirochetes. On the day of harvest, which varied between donor-specific mouse batches due to their health status (see Tables 1 and 2; Fig. 1), ear pinna, heart, tibiotarsal joint, and urinary bladder tissues were aseptically harvested from each challenged mouse and subjected to cultivation in BSK-II medium. Specifically, both sides of the sagittally dissected heart from each mouse were cultured in an 8 mL polystyrene tube (Becton Dickinson, NJ, USA) containing 3 mL of BSK-II medium. The other tissues of each mouse (a urinary bladder dissected in half, a piece of ear pinna of ~2–3 mm in diameter, and a tip of a tibiotarsal joint) were cultivated in a 1.7 mL polypropylene microcentrifuge tube (Denville Scientific, MA, USA) with 1 mL of BSK-II medium. Tissue cultures were checked using DFM weekly for up to 4 weeks. To demonstrate the infectivity phenotypes of the wild type and its isogenic mutant, C3H/HeJ (C3H) mice (7–8 weeks of age; the Jackson Laboratory) were s.c. challenged with a 100 μL inoculum (~1 × 10^4^ spirochetes per mouse) of B31 or ∆VlsE (three males and three females per strain) within the shoulder area. Tables 1 and 2 and Fig. 1 indicate the numbers of humanized mice per each group and the respective experimental timelines.

**TABLE 1:** Culture results of tissues harvested from UCB-HSC NSG and C3H mice challenged with *B. burgdorferi* B31 and ∆VlsE

Tissue harvested for culture upon sacrifice	No. of cultures positive/total no. of tested				
	UCB-HSC NSG			C3H	
	B31 (Group I)	∆VlsE (Group II)	∆VlsE + LPS^*c*^ (Group III)	B31 (Group I)	∆VlsE (Group II)
Blood (day 7 pc^*a*^)	6/6	7/7	7/7	6/6	6/6
Bladder (day 27/41 pc^*b*^)	5/6	6/7	5/7	6/6	0/6
Ear pinna (day 27/41 pc)	6/6	4/7	6/7	6/6	0/6
Heart (day 27/41 pc)	6/6	5/7	7/7	6/6	0/6
Tibiotarsal joint (day 27/41 pc)	6/6	5/7	7/7	6/6	0/6
Total infected (day 27/41 pc)	6/6	7/7	7/7	6/6	0/6

^*a*^ pc, postchallenge.

^*b*^ The humanized mouse data were combined from two biological replicates, and the days of sacrifice were 27 and 41 postchallenge. C3H mice were sacrificed at day 21 postchallenge.

^*c*^ LPS, lipopolysaccharide.

**TABLE 2:** Culture results of tissues harvested from huNOG-EXL SA and C3H mice challenged with *B. burgdorferi* B31 and ∆VlsE

Tissue harvested for culture upon sacrifice	No. of cultures positive/total no. of tested				
	huNOG-EXL SA			C3H	
	B31 (Group I)	∆VlsE (Group II)	∆VlsE + LPS^*c*^ (Group III)	B31 (Group I)	∆VlsE (Group II)
Blood (day 7 pc^*a*^)	5/5	6/6	4/4	6/6	6/6
Bladder (day 21/41 pc^*b*^)	5/5	3/6	3/4	6/6	0/6
Ear pinna (day 21/41 pc)	5/5	4/6	2/4	6/6	0/6
Heart (day 21/41 pc)	5/5	5/6	3/4	6/6	0/6
Tibiotarsal joint (day 21/41 pc)	4/5	5/6	4/4	6/6	0/6
Total infected (day 21/41 pc)	5/5	6/6	4/4	6/6	0/6

^*a*^ pc, postchallenge.

^*b*^ The humanized mouse data were combined from two biological replicates, and the days of sacrifice were 21 and 41 postchallenge. C3H mice were sacrificed at day 21 postchallenge.

^*c*^ LPS, lipopolysaccharide.

### Immunization of mice with RECOMBITEK Lyme vaccine and wild-type challenge

To validate the efficacy of the RECOMBITEK Lyme vaccine (Boehringer-Ingelheim, Germany), we performed a pilot study in which eight male and eight female C3H mice (7–8 weeks of age) were acquired from the Jackson Laboratories. After an adaptation period, five males and five females of group A were intramuscularly (i.m.) immunized with 100 μL (50 μL per hind limb) of RECOMBITEK Lyme vaccine at days 0 and 28. After 2 weeks post-second immunization, each of group A animals and infectivity control mice (three males and three females) was s.c. challenged with a 100 μL inoculum of B31 (~1 × 10^5^ spirochetes per mouse) in the shoulder area. To assess the outcome of challenge and vaccination, all mice were sacrificed at day 21 pc and ear pinna, heart, tibiotarsal joint, and urinary bladder tissues harvested from each animal were cultured as described above.

**Fig 2:**
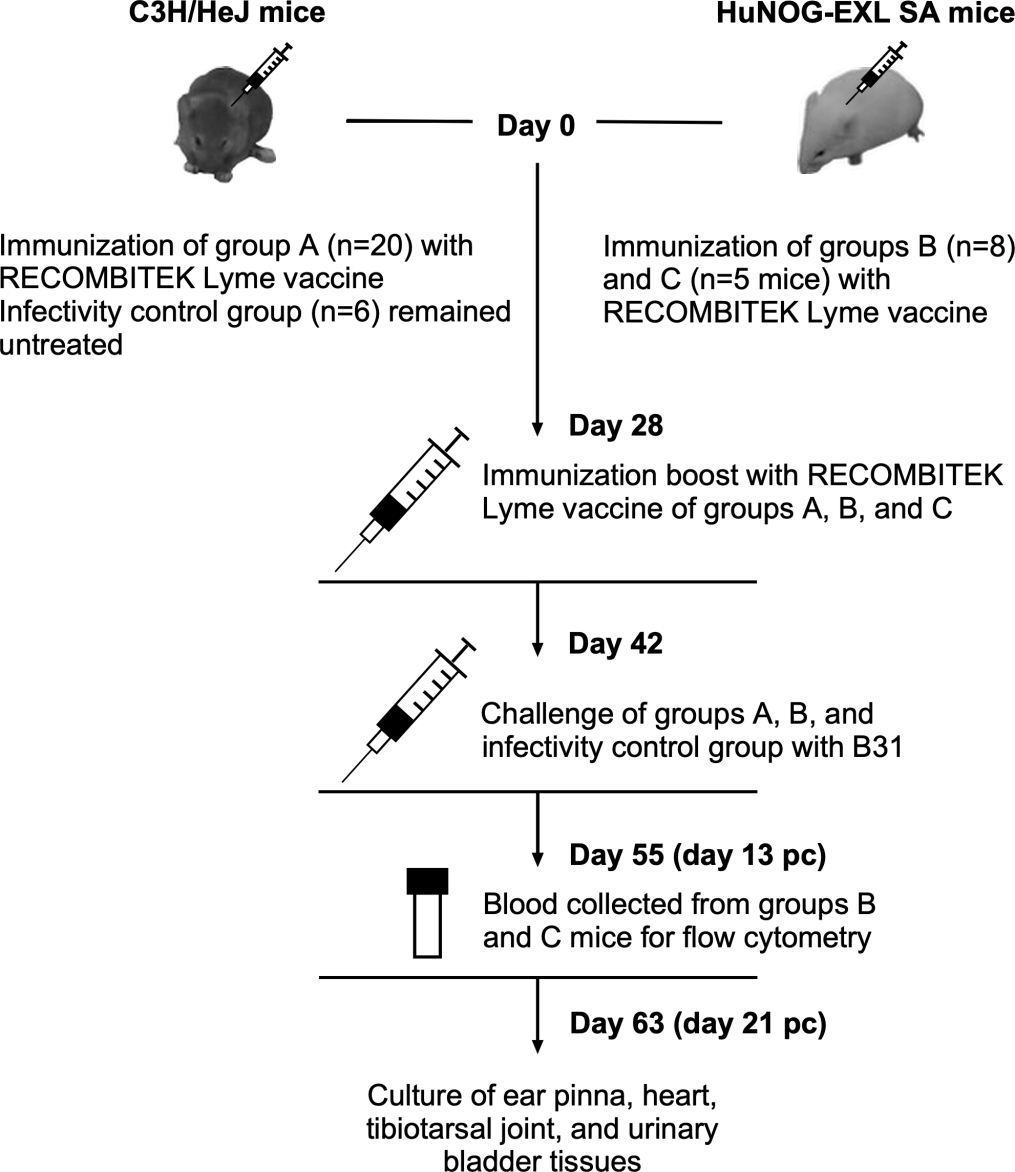
The experimental design of the vaccination study assessing humanized mice for their immune responsiveness to a single antigen of *B. burgdorferi*. Group A (C3H/HeJ), group B (huNOG-EXL SA), and group C (huNOG-EXL SA) were intramuscularly immunized with the RECOMBITEK Lyme vaccine at days 0 and 28. The infectivity control group (C3H/HeJ) received no vaccination. The group sizes are indicated in the figure. At day 42, groups A and B mice and infectivity control animals were subcutaneously challenged with *B. burgdorferi* B31-A3 (B31). At day 55 (day 13 postchallenge (pc)), blood was collected from all humanized mice (groups B and C) for flow cytometry analysis. All huNOG-EXL SA and C3H/HeJ mice were, respectively, euthanized at day 63 (day 21 pc). Ear pinnae, hearts, tibiotarsal joints, and urinary bladders collected from each challenged mouse were cultured to evaluate the outcome of vaccination.

Following the success of the pilot study, 5 male and 5 female C3H mice and 13 huNOG-EXL SA females were i.m. immunized with RECOMBITEK Lyme vaccine as detailed above. At day 42, 8 HuNOG-EXL SA and 10 C3H mice were s.c. challenged with a 100 μL inoculum of B31 (~1 × 10^5^ spirochetes per mouse) in the shoulder area. At day 13 pc, ~120 µL of blood was collected from each of the huNOG-EXL SA mice via cheek bleed into lithium-heparin tubes (Becton Dickinson) and subjected to flow cytometry analysis within 16 h. At day 21 pc, ear pinna, heart, tibiotarsal joint, and urinary bladder tissues were cultured from each animal as detailed above (Fig. 2).

### Flow cytometry

During the sacrifice, blood samples (~120 µL each) from 76 mice consisting of 22 groups total were collected via cheek bleed into heparin tubes (Becton Dickinson) and analyzed for human immune cells (hCD45, CD19, CD3, CD4, CD8, and CD33) within <16 h by flow cytometry using the BD Biosciences LSRII or FACSCalibur instrument (Becton Dickinson) as detailed (81). The flow cytometry data were analyzed using FlowJo (Tree Star, OR, USA). All human monoclonal antibodies and corresponding controls were obtained from BD PharMingen (CA, USA) and conjugated with FITC, PE, PerCP, APC, Alexa 405, Pacific Blue, or Alexa 700. The representative flow cytometry data showing the gating strategy are provided in Fig. S4.

### Western blot

B31 was grown in BSK-II medium to the late stationary phase. Cells were counted, pelleted by centrifugation at 6,000 × *g* for 10 min at 4°C, and then washed twice with ice-cold phosphate-buffered saline (PBS; Sigma-Aldrich, MO, USA). After washing, cells were prepared by mixing with Protein Loading Buffer Blue (National Diagnostics, MI, USA). Cells were lysed by incubating them at 95°C for 5 min. Then, 20 μL of cellular mixtures (~1 × 10^7^ cells) loaded per lane on Novex 8–16% Tris-Glycine Mini Protein Gel (WedgeWell format, ThermoFisher Scientific, MA, USA) were separated by sodium dodecyl sulfate-polyacrylamide gel electrophoresis (SDS-PAGE) using a Mini Gel Tank and PowerEase Touch 120 W Power Supply (ThermoFisher Scientific). Samples were transferred on both polyvinylidene fluoride (PVDF) and nitrocellulose iBlot 3 transfer stacks (ThermoFisher Scientific) using the iBlot 3 Gel Transfer Device (ThermoFisher Scientific). After transfer, membranes were cut into strips (two lanes of B31 proteins per strip) and blocked for 1 h with 5% non-fat milk (Bio-Rad, MI, USA) prepared in Tris-buffered saline (TBS; Bio-Rad).

For each mouse group, sera were pooled to a final volume of 250 µL (63–125 µL per mouse; group size varied due to mortality; see legends of Fig. S1 to S3 for more details). Pooled mouse sera and individual human blood samples were mixed with 5 mL of TBS supplemented with 5% non-fat milk (1:20) according to a previously published protocol designed to detect anti-*Bb* humoral immune response (82), and then transferred to 60 × 5 mm Petri dishes (Corning, NY, USA). All membrane strips were incubated overnight at 4°C. Three human whole blood samples were received from the Lyme Disease Biobank in ethylenediaminetetraacetic acid (EDTA) tubes (83) and each independently served as a positive control for both anti-borrelia IgM and IgG antibodies. For the positive control incubations, 250 µL of whole blood mixed with 5 mL of TBS supplemented with 5% non-fat milk was used to incubate the membrane strips. Following the initial incubation, the membrane strips were washed four times for 10 min each in TBS. Each strip was then cut in half and incubated for 1 h at room temperature with either goat anti-human IgG (H+L) or goat anti-human IgM (H+L) horseradish peroxidase secondary antibodies (both 1:40,000; ThermoFisher Scientific) and subsequently washed four times for 10 min with TBS. Membranes were imaged on a ChemiDoc imaging system (Bio-Rad) using enhanced chemiluminescence (ECL) Western Blotting Substrate (ThermoFisher Scientific).

### Statistics

All statistical analyses were performed using R (version 4.5.0 [84]); with the lme4 and emmeans packages (84, 85). Categorical variables were summarized using frequencies and percentages. Linear mixed-effects models were used to evaluate characterization of human immune cells with experiment batch as random intercept (86). Model assumptions were assessed using residual plots. Pairwise comparisons were performed among groups with Tukey’s adjustment for *P* values. A *P* value of 0.05 was considered significantly different.

## RESULTS

### Humanized mice fail to clear an antibody-susceptible *B. burgdorferi* strain

To examine whether humanized mouse models, UCB-HSC NSG and huNOG-EXL SA, had the capacity to mount a protective anti-*Bb* antibody response, mice of each model were broken down into five groups. Group I animals were challenged with B31, and mice of groups II and III were inoculated with its isogenic mutant, ∆VlsE. Groups IV and V mice remained intact (Fig. 1). At day 7 pc, blood was taken from each challenged animal to confirm the expected *Bb* infection via culture. Both B31 and ∆VlsE were previously shown to consistently establish culture-detectable spirochetemia at this time point (28–31). As anticipated, spirochetes were detected in day-7 blood samples of all challenged humanized (groups I, II, and III) and C3H mice. To additionally stimulate the humanized immune system, group III ∆VlsE-infected mice and naïve controls (group IV) were injected with LPS at day 7 pc. On the day of harvest, which considerably varied between the groups for each model because of their donor-dependent health status, all challenged humanized mice tested positive by culture regardless of *Bb* strain used or LPS treatment applied (Tables 1 and 2). We expectedly confirmed the disseminated (systemic) infection by wild-type B31, which had an intact VlsE-mediated antigenic variation system that would allow wild-type spirochetes to evade *Bb* antibodies in immunocompetent mice (28–31). Importantly, the establishment of systemic ∆VlsE infection, together with the respective negative culture results of ∆VlsE-challenged C3H controls, clearly demonstrated the lack of capacity of the tested humanized models to achieve antibody-mediated clearance under our experimental conditions.

### OspA vaccination fails to protect huNOG-EXL SA mice from *B. burgdorferi* B31 challenge

It is possible that challenging humanized mice with ∆VlsE presented too many antigens to the humanized immune system potentially overwhelming it. To overcome this plausible scenario, we decided to stimulate the humanized immune system with a single *Bb* antigen. For that, we took advantage of commercially available efficacious canine LD vaccine, RECOMBITEK, whose nonadjuvanted formulation included large complexes of lipidated outer surface protein A (OspA) (87, 88). Mice in groups A and B were immunized twice with RECOMBITEK at a 4-week interval, followed by challenge of both groups and the respective infectivity control group with B31 (Fig. 2). The culture results showed that all six infectivity control mice became infected, indicating that the B31 inoculum used to challenge the immunized animals was fully infectious (Table 3). The data of experimental group A demonstrated a 70% protection rate for both C3H males and females. In contrast, none of the vaccinated huNOG-EXL SA mice (*n* = 8) were protected from B31 challenge, indicating the failure of the humanized immune system to achieve OspA-vaccine-mediated protection in this model.

**TABLE 3:** Culture results of tissues harvested from huNOG-EXL SA and C3H mice treated with OspA vaccine and challenged with *B. burgdorferi* B31

Tissue harvested at day 21 pc^*a*^	No. of cultures positive/total no. of tested				
	Vaccinated			Unvaccinated	
	Group A		Group B	Infectivity control	
	C3H female	C3H male	huNOG-EXL SA	C3H female	C3H male
Bladder	2/10	3/10	7/8	3/3	3/3
Ear pinna	2/10	1/10	6/8	3/3	3/3
Heart	2/10	1/10	7/8	3/3	3/3
Tibiotarsal joint	2/10	2/10	5/8	2/3	1/3
Total infected	3/10	3/10	8/8	3/3	3/3
Total protected	7/10	7/10	0/8	n/a^*b*^	n/a^*b*^

^*a*^ pc, postchallenge.

^*b*^ n/a, non-applicable.

### Flow cytometry and Western blot provide no evidence of anti-*Bb* antibody response in humanized mice

To test whether UCB-HSC NSG and huNOG-EXL SA mice developed any anti-*Bb* human IgM and IgG antibodies upon B31 or ∆VlsE challenge, we collected sera for Western blot (WB) analysis prior to each sacrifice (see Fig. 1 and 2 for days of collection). While sera from all three human LD-confirmed patients consistently showed the anticipated reactivity to *Bb* antigens, the overall WB results were not interpretable as the similar reactivity was also observed across all negative controls (Fig. S1 to S3), indicating the poly-reactive nature of humanized mouse antibodies. Due to unexpected mortality and limited terminal blood volumes collected, the amounts of sera from RECOMBITEK-immunized huNOG-EXL SA mice were insufficient for quantifying OspA-specific antibody titers.

**Fig 3:**
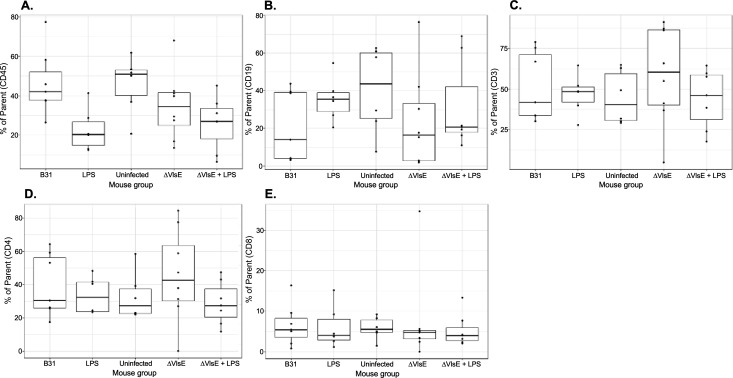
The percentage of human leukocyte subsets in peripheral blood collected from *B. burgdorferi*-infected and control UCB-HSC NSG mice. The box-and-whisker plot shows the median (line), interquartile range (box), whiskers to values within 1.5 × interquartile range, and overlaid individual data points. Percent of parent lymphocyte population frequencies was quantified by flow cytometry using peripheral blood collected at days 27 (15 mice; donors HuCB 1957.2 and HuCB 1957.3) and 35 (19 mice; donor HuCB 1946.1). Panels show the frequencies of hCD45^+^ cells (panel **A**), CD19^+^ B cells (panel **B**), CD3^+^ T cells (panel **C**), CD4^+^ T cells (panel **D**), and CD8^+^ T cells (panel **E**). B31 denotes the mice that were infected with *B. burgdorferi* B31-A3 (four and three samples from donors HuCB 1946.1 and HuCB 1957.2, respectively). LPS denotes the uninfected mice that were treated with lipopolysaccharide (three samples from each donor, HuCB 1946.1 and HuCB 1957.2). Uninfected denotes the uninfected and untreated mice (four and two samples from donors HuCB 1946.1 and HuCB 1957.3, respectively). ∆VlsE denotes the mice that were infected with *B. burgdorferi* B31-A3∆*vls* (four samples from each donor, HuCB 1946.1 and HuCB 1957.2). ∆VlsE + LPS denotes the mice that were infected with ∆VlsE and treated with LPS (four and three samples from donors HuCB 1946.1 and HuCB 1957.2, respectively).

**Fig 4:**
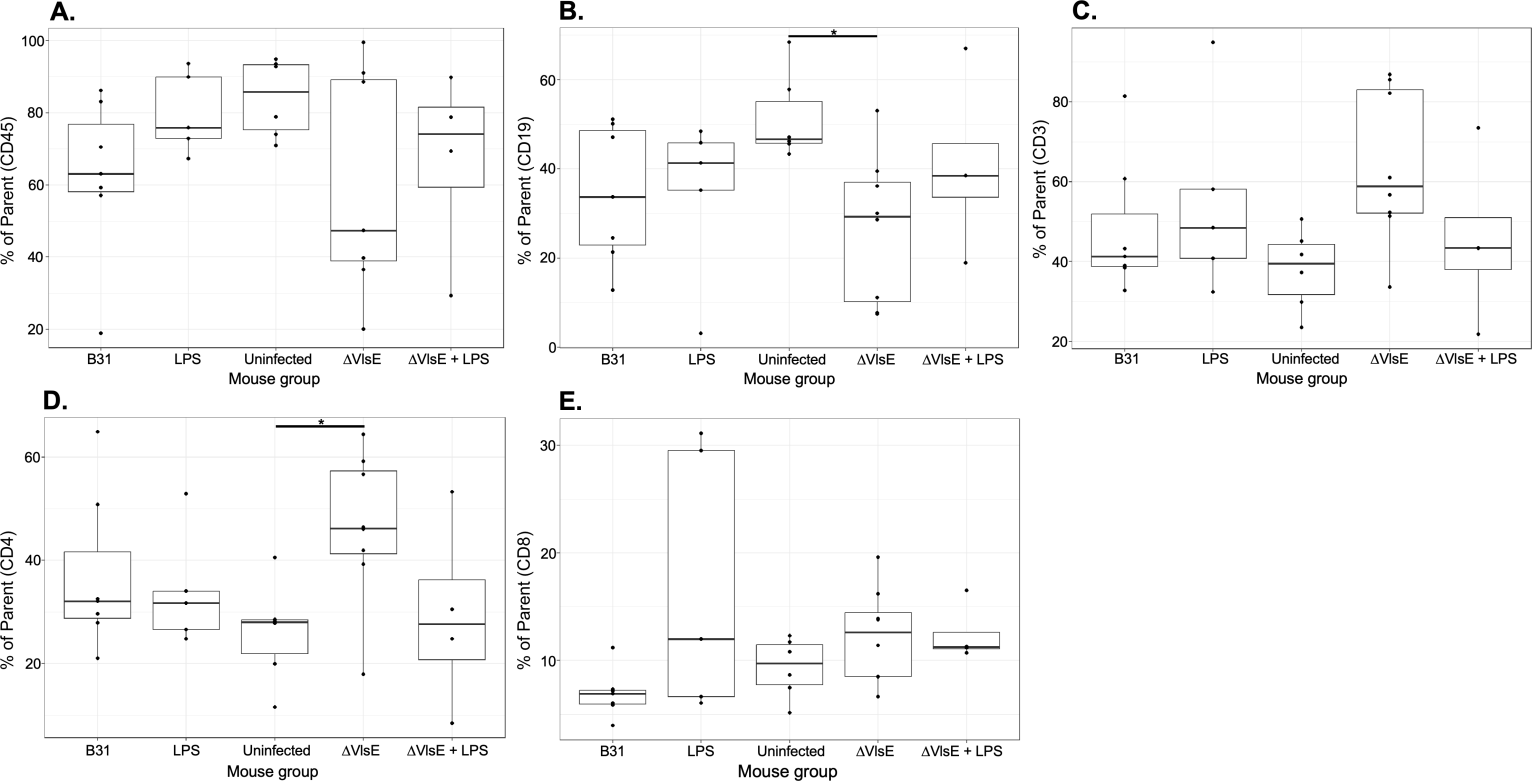
The percentage of human leukocyte subsets in peripheral blood collected from *B. burgdorferi*-infected and control huNOG-EXL SA mice. The box-and-whisker plot shows the median (line), interquartile range (box), whiskers to values within 1.5 × interquartile range, and overlaid individual data points. Percent of parent lymphocyte population frequencies was quantified by flow cytometry using peripheral blood collected at days 35 (3 and 12 samples from donors 55 and 56, respectively) and 21 (15 samples from donor 007). Panels show the frequencies of hCD45^+^ cells (panel **A**), CD19^+^ B cells (panel **B**), CD3^+^ T cells (panel **C**), CD4^+^ T cells (panel **D**), and CD8^+^ T cells (panel **E**). B31 denotes the mice that were infected with *B. burgdorferi* B31-A3 (three, one, and three samples from donors 007, 55, and 56, respectively). LPS denotes the uninfected mice treated with lipopolysaccharide (three and two samples from donors 007 and 56, respectively). Uninfected denotes the untreated and uninfected mice (three samples from each donor, 007 and 56). ∆VlsE denotes the mice that were infected with *B. burgdorferi* B31-A3∆*vls* (three, two, and three samples from donors 007, 55, and 56, respectively). ∆VlsE + LPS denotes the mice that were infected with ∆VlsE and treated with LPS (three and one samples from donors 007 and 56, respectively). The asterisks denote a statistical significance (*P* = 0.045 in panel B and *P* = 0.0294 in panel D).

**Fig 5:**
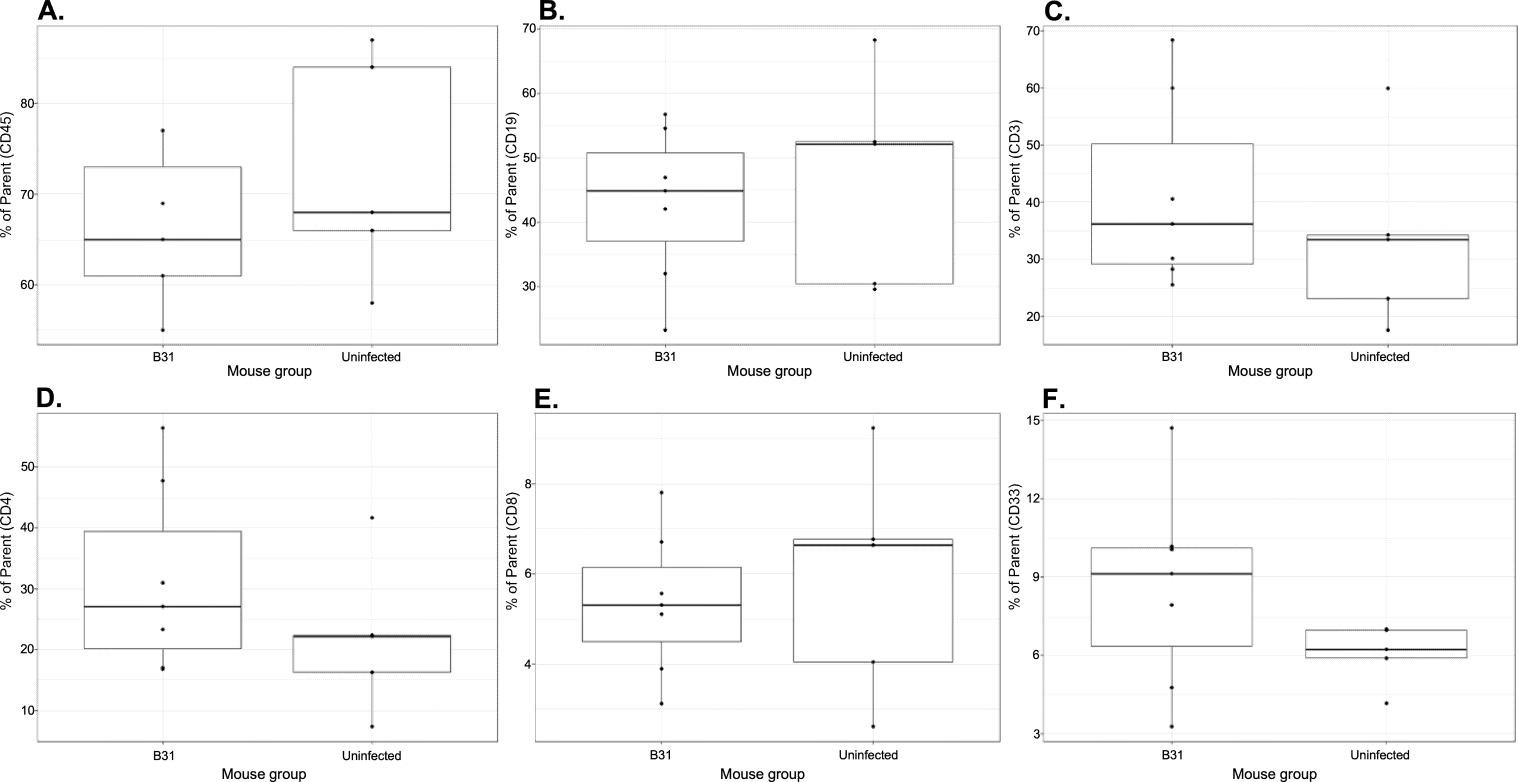
The percentage of human leukocyte subsets in peripheral blood collected from Lyme disease vaccine-treated huNOG-EXL SA mice. The box-and-whisker plot shows the median (line), interquartile range (box), whiskers to values within 1.5 × interquartile range, and overlaid individual data points. Percent of parent lymphocyte population frequencies was quantified by flow cytometry using peripheral blood collected at day 55 (13 days postchallenge; 12 samples from donor 127). Panels show the frequencies of hCD45^+^ cells (panel **A**), CD19^+^ B cells (panel **B**), CD3^+^ T cells (panel **C**), CD4^+^ T cells (panel **D**), CD8^+^ T cells (panel **E**), and myeloid cells (panel **F**) cells. B31 denotes the mice (*n* = 7) that were vaccinated twice with 100 μL of RECOMBITEK Lyme vaccine per mouse at days 0 and 28, and challenged at day 42 with *B. burgdorferi* B31-A3. Uninfected denotes the uninfected control mice vaccinated twice with 100 μL of RECOMBITEK Lyme and otherwise not challenged (*n* = 5).

In addition to the WB analysis, we also collected blood samples to quantify human leukocyte subsets (hCD45^+^, CD19^+^ B, CD3^+^ T, CD4^+^ T, and CD8^+^ T cells) in the peripheral blood of all groups of UCB-HSC NSG and huNOG-EXL SA mice, including those of the vaccination study (Fig. 3 to 5). Overall, the results demonstrated no statistical difference between the groups of each experiment for any model (Fig. 3 to 5), indicating the lack of immune responsiveness to *Bb* infection. Two exceptions were significantly lower and higher frequencies of CD19^+^ B cells (*P* = 0.045) and CD4^+^ T cells (*P* = 0.0294), respectively, when the ∆VlsE-infected huNOG-EXL SA mice were compared to the respective uninfected controls (Fig. 4, panels B and D).

## DISCUSSION

In the current investigation, we assessed two humanized mouse models, UCB-HSC NSG and huNOG-EXL SA, for their potential as bridging models for testing LD vaccine candidates. Our two tested models were generated through engraftment of UCB-derived HSC and represented the best available models provided by the developers. Transplantation with UCB was shown to yield a more efficient engraftment than injection with PBMCs, although both types of reconstitution support production of human IgM and/or IgG (89–93). NSG mice intrahepatically engrafted with human fetal liver (HFL) or UCB-derived HSC were reported to substantially increase plasma levels of human IgM and IgG when animals were immunized with keyhole limpet hemocyanin (91). PBMC-engrafted NSG mice immunized against a chimeric (Hepatitis B virus) antigen produced both IgM and IgG with some having the infection-neutralizing capacity (93). Furthermore, engraftment of NSG-SGM3 mice with bone marrow-liver-thymus (BLT) also resulted in generating (dengue virus) antigen-specific human IgM and IgG antibodies (94). Consistently, huNOG-EXL mice also demonstrated significantly elevated levels of serum IgG 16 weeks post-engraftment (95). However, in another study, CD34-engrafted NOG-EXL mice could only generate IgM against *Pneumocystis murina* antigens with no detectable level of IgG (66).

For our study, the tested models were generated by using multiple human donors, which allowed a greater objectivity and genetic diversity during mouse testing. The experimental design consisted of two parts with distinct but complementary aims. The first set of experiments aimed to assess the capacity of the humanized immune system to develop protective antibodies when exposed to multiple *Bb* antigens. To achieve it, we made use of previously generated *Bb* mutant, ∆VlsE, which, due to the genetic deletion of its antigenic variation system, was susceptible to acquired anti-*Bb* mouse antibodies. It was repeatedly shown that ∆VlsE was cleared by the adaptive antibody-mediated response of C3H mice within 2–3 weeks pc (28–31). Moreover, C3H mice that had cleared ∆VlsE infection fully abrogated B31 reinfection (31). Finally, anti-∆VlsE antibodies passively transferred to immunodeficient mice efficiently prevented the tick- or needle-mediated B31 challenge (30). Therefore, in this study, we considered and utilized the ∆VlsE mutant as an efficacious live-attenuated vaccine strain. The aim of the second study was to evaluate humanized mice (huNOG-EXL SA) for their ability to induce a protective anti-*Bb* antibody response upon OspA vaccination. The rationale of this experiment was to stimulate the humanized immune system with a single *Bb* antigen as opposed to overburdening it with hundreds of ∆VlsE antigens. For this, we used an OspA antigen in the form of a transmission-blocking canine vaccine (RECOMBITEK) that proved its high efficacy in dogs (87, 88). It is also worth noting that the OspA antigen was used to constitute an efficacious human vaccine (LYMErix) that was only available to the U.S. public between December 1998 and February 2002 (96). Of note, even to date, in ongoing efforts to develop a safe human LD vaccine, OspA is still actively tested as the top target (97–100). In contrast to the demonstrated capacity of the immunocompetent mouse immune system to develop protective anti-*Bb* antibodies upon ∆VlsE or OspA immunization, the culture results of challenged huNOG-EXL SA mice confirmed the incapacity of the humanized immune system to provide antibody-mediated protection against *Bb*.

Western blot of pooled sera from UCB-HSC NSG or huNOG-EXL SA mice yielded non-specific bands indicating the poly-reactive nature of humanized antibodies. This is consistent with a previous study where approximately a quarter of mature naïve B cells from NSG mice reconstructed with human fetal and bone marrow were poly-reactive (101). Moreover, human IgM antibodies produced in BLT-NSG mice actively infected with dengue virus were highly cross-reactive as well (102). The poly-reactive nature of humanized antibodies and limited amounts of sera collected from mice prevented us from further characterization of anti-*Bb* immune response (e.g., measurement of OspA-specific antibody titers). In addition to measuring anti-OspA antibodies, future studies are warranted to examine whether the lack of immune responsiveness to *Bb* infection in the tested humanized models (Fig. 3 to 5) could be accounted for by the previously shown ability of the Lyme pathogen to suppress immune responses (103, 104).

Although our results do not justify using the humanized models examined in the present study for the preclinical testing of LD vaccine candidates, the potential utility of humanized mice for LD research should not be discarded. By utilizing *Bb*-infected NSG mice, a recent study showed that anti-*Bb* antibody response was modulated by human Fc*γ*RIIb receptor, which in turn supposedly diminished the level of self-reactive antibodies (82). Furthermore, future studies are still warranted to test any newly developed or to-be-developed humanized model for the capacity to develop an effective anti-*Bb* sterilizing immune response. For example, it would be worth examining a recently developed humanized THX mice, as the model was shown to develop mature T-cell-dependent antibody responses that involved both class-switching plasma and memory B cells (94). Unfortunately, our attempts to acquire this new model for our study were not successful. Finally, in the context of our present findings, it is important to re-emphasize that given the extracellular nature of the LD spirochete and that acquired antibodies are the primary effector mechanism of anti-*Bb* immune response, all currently existing *in vitro* systems (e.g., human primary and transformed cell lines and organoids [105–111]) that have been elegantly utilized to learn about the borrelial pathogenesis cannot substitute classical mouse models for either preclinical LD vaccine or *Bb* pathogenesis (e.g., arthritis and carditis) investigations.

## SUPPLEMENTAL MATERIAL

Supplemental Material spectrum.03803-25-s0001.pdf Supplemental tables and figures.

ASM does not own the copyrights to Supplemental Material that may be linked to, or accessed through, an article. The authors have granted ASM a non-exclusive, world-wide license to publish the Supplemental Material files. Please contact the corresponding author directly for reuse.
