# Supplementary material for: Testing the capacity of humanized immune system mice to induce a protective antibody response against the Lyme disease pathogen
Source: Microbiol Spectr. 2026 Apr 7;14(5):e03803-25. doi: 10.1128/spectrum.03803-25 (PMC13142037; doi:10.1128/spectrum.03803-25)
Supplement: Supplemental Material — Supplemental tables and figures. [file spectrum.03803-25-s0001.pdf]

SUPPLEMETAL MATERIALS

Supplemental Tables

Table S1. The ages of UCB-HSC NSG mice at the time of *Borreliella burgdorferi* challenge and sacrifice.

Sex	Date of birth	Date of engraftment	Age at day 0	Age at the time of sacrifice
**Donor HuCB 1946.1**				
Female	10/26/21	12/01/21	23 weeks	28 weeks 6 days
Female	10/26/21	12/01/21	23 weeks	28 weeks 6 days
Female	10/26/21	12/01/21	23 weeks	28 weeks 6 days
Female	10/26/21	12/01/21	23 weeks	28 weeks 6 days
Female	10/26/21	12/01/21	23 weeks	28 weeks 6 days
Female	10/26/21	12/01/21	23 weeks	28 weeks 6 days
Female	10/26/21	12/01/21	23 weeks	28 weeks 6 days
Female	10/26/21	12/01/21	23 weeks	28 weeks 6 days
Female	10/26/21	12/01/21	23 weeks	28 weeks 6 days
Female	10/26/21	12/01/21	23 weeks	28 weeks 6 days
Female	10/26/21	12/01/21	23 weeks	28 weeks 6 days
Female	10/26/21	12/01/21	23 weeks	28 weeks 6 days
Female	10/26/21	12/01/21	23 weeks	28 weeks 6 days
Female	10/26/21	12/01/21	23 weeks	28 weeks 6 days
Female	10/26/21	12/01/21	23 weeks	28 weeks 6 days
Female	10/26/21	12/01/21	23 weeks	28 weeks 6 days
Female	10/26/21	12/01/21	23 weeks	28 weeks 6 days
Female	10/26/21	12/01/21	23 weeks	28 weeks 6 days
Female	10/26/21	12/01/21	23 weeks	28 weeks 6 days
**Donor HuCB 1957.2**				
Female	12/21/21	01/21/22	31 weeks 6 days	36 weeks
Female	12/21/21	01/21/22	31 weeks 6 days	36 weeks
Female	12/21/21	01/21/22	31 weeks 6 days	36 weeks
Female	12/21/21	01/21/22	31 weeks 6 days	36 weeks
Female	12/21/21	01/21/22	31 weeks 6 days	36 weeks
Female	12/21/21	01/21/22	31 weeks 6 days	36 weeks
Female	12/21/21	01/21/22	31 weeks 6 days	36 weeks
Female	12/21/21	01/21/22	31 weeks 6 days	36 weeks

Female	12/21/21	01/21/22	31 weeks 6 days	36 weeks
Female	12/21/21	01/21/22	31 weeks 6 days	36 weeks
Female	12/21/21	01/21/22	31 weeks 6 days	36 weeks
Female	12/21/21	01/21/22	31 weeks 6 days	36 weeks
Female	12/21/21	01/21/22	31 weeks 6 days	36 weeks
**Donor HuCB 1957.3**				
Female	12/21/21	01/21/22	31 weeks 6 days	36 weeks
Female	12/21/21	01/21/22	31 weeks 6 days	36 weeks

Table S2. The ages of huNOG-EXL SA mice at the time of *Borreliella burgdorferi* challenge and sacrifice.

Sex	Week of birth	Week of engraftment	Age at day 0	Age at the time of sacrifice
**Donor 055**				
Female	07/12/22	08/23/22	25 weeks 6 days	31 weeks 5 days
Female	07/12/22	08/23/22	25 weeks 6 days	31 weeks 5 days
Female	07/12/22	08/23/22	25 weeks 6 days	31 weeks 5 days
**Donor 056**				
Female	07/13/22	08/24/22	25 weeks 5 days	31 weeks 4 days
Female	07/13/22	08/24/22	25 weeks 5 days	31 weeks 4 days
Female	07/13/22	08/24/22	25 weeks 5 days	31 weeks 4 days
Female	07/13/22	08/24/22	25 weeks 5 days	31 weeks 4 days
Female	07/13/22	08/24/22	25 weeks 5 days	31 weeks 4 days
Female	07/13/22	08/24/22	25 weeks 5 days	31 weeks 4 days
Female	07/13/22	08/24/22	25 weeks 5 days	31 weeks 4 days
Female	07/13/22	08/24/22	25 weeks 5 days	31 weeks 4 days
Female	07/13/22	08/24/22	25 weeks 5 days	31 weeks 4 days
Female	07/13/22	08/24/22	25 weeks 5 days	31 weeks 4 days
Female	07/13/22	08/24/22	25 weeks 5 days	31 weeks 4 days
Female	07/13/22	08/24/22	25 weeks 5 days	31 weeks 4 days
**Donor 007**				
Female	11/30/22	01/11/23	20 weeks 6 days	25 weeks 1 day
Female	11/30/22	01/11/23	20 weeks 6 days	25 weeks 1 day
Female	11/30/22	01/11/23	20 weeks 6 days	25 weeks 1 day
Female	11/30/22	01/11/23	20 weeks 6 days	25 weeks 1 day
Female	11/30/22	01/11/23	20 weeks 6 days	25 weeks 1 day
Female	11/30/22	01/11/23	20 weeks 6 days	25 weeks 1 day
Female	11/30/22	01/11/23	20 weeks 6 days	25 weeks 1 day
Female	11/30/22	01/11/23	20 weeks 6 days	25 weeks 1 day
Female	11/30/22	01/11/23	20 weeks 6 days	25 weeks 1 day
Female	11/30/22	01/11/23	20 weeks 6 days	25 weeks 1 day
Female	11/30/22	01/11/23	20 weeks 6 days	25 weeks 1 day
Female	11/30/22	01/11/23	20 weeks 6 days	25 weeks 1 day
Female	11/30/22	01/11/23	20 weeks 6 days	25 weeks 1 day
Female	11/30/22	01/11/23	20 weeks 6 days	25 weeks 1 day

Female	11/30/22	01/11/23	20 weeks 6 days	25 weeks 1 day
**Donor 127**				
Female	08/08/23	09/19/23	28 weeks 2 days	31 weeks 1 day
Female	08/08/23	09/19/23	28 weeks 2 days	31 weeks 1 day
Female	08/08/23	09/19/23	28 weeks 2 days	31 weeks 1 day
Female	08/08/23	09/19/23	28 weeks 2 days	31 weeks 1 day
Female	08/08/23	09/19/23	28 weeks 2 days	31 weeks 1 day
Female	08/08/23	09/19/23	28 weeks 2 days	31 weeks 1 day
Female	08/08/23	09/19/23	28 weeks 2 days	31 weeks 1 day
Female	08/08/23	09/19/23	28 weeks 2 days	31 weeks 1 day
Female	08/08/23	09/19/23	28 weeks 2 days	31 weeks 1 day
Female	08/08/23	09/19/23	28 weeks 2 days	31 weeks 1 day
Female	08/08/23	09/19/23	28 weeks 2 days	31 weeks 1 day
Female	08/08/23	09/19/23	28 weeks 2 days	31 weeks 1 day
Female	08/08/23	09/19/23	28 weeks 2 days	31 weeks 1 day

Supplemental Figure

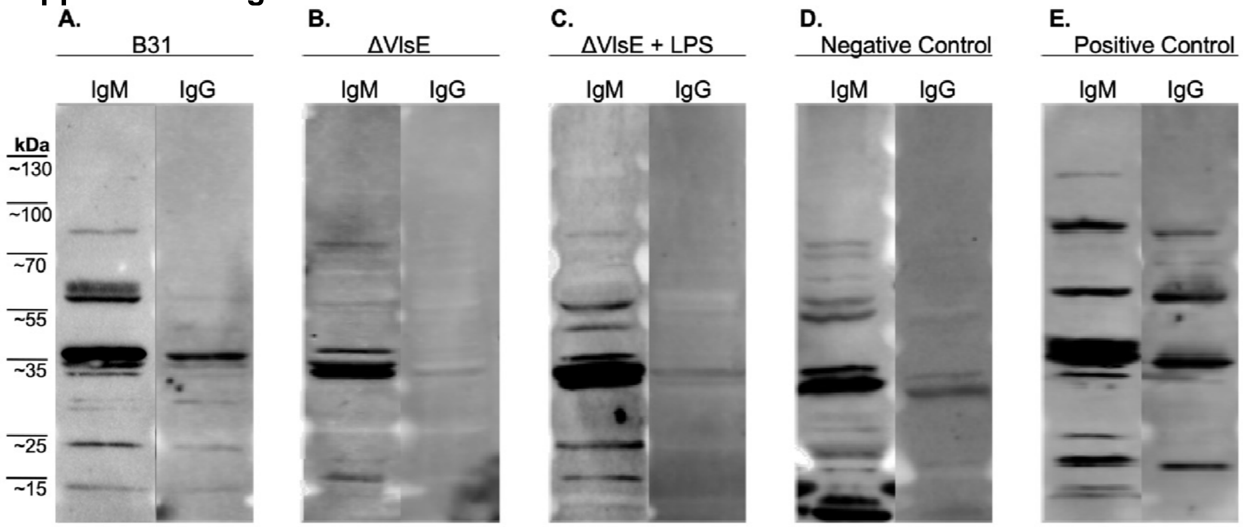

Figure S1. Immunoblots of sera pooled from UCB-HSC NSG mice (donor HuCB 1946.1) and probed against whole-cell lysates of *Borreliella burgdorferi*. Pooled sera from UCB-HSC NSG mice (collected at day 41 postchallenge from donor HuCB 1946.1) infected with *B. burgdorferi* B31-A3 (B31; pooled from 4 mice; panel A), B31-A3Δ*vls* (ΔVlsE; pooled from 4 mice; panel B), or treated with lipopolysaccharide (LPS) and infected with ΔVlsE (pooled from 4 mice; panel C) were probed against whole-cell lysates of B31 (~10^7 cells per lane). Sera pooled from 4 uninfected UCB-HSC NSG mice (donor HuCB 1946.1) served as a negative control (panel D). A human whole blood sample received from the Lyme Disease Biobank served as a positive control for both anti-borrelia IgM and IgG antibodies (panel E).

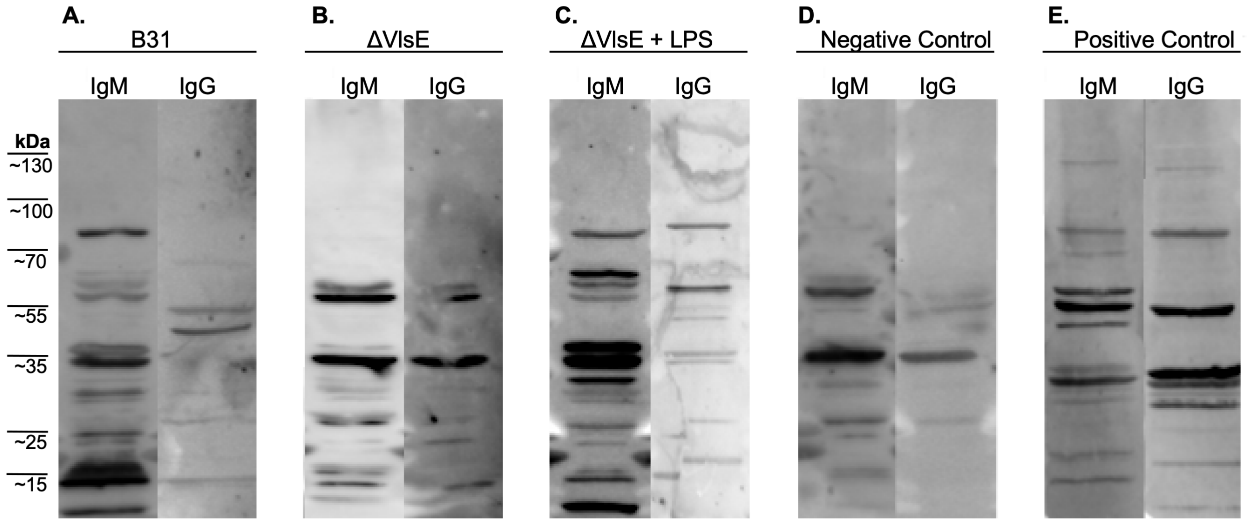

Figure S2. Immunoblots of sera pooled from UCB-HSC NSG mice (donors HuCB 1957.2 and HuCB 1957.3) and probed against whole-cell lysates of *Borreliella burgdorferi*. Pooled sera from UCB-HSC NSG mice (collected at day 27 postchallenge from donors HuCB 1957.2 and HuCB 1957.3) infected with *B. burgdorferi* B31-A3 (B31; pooled from one mouse of each donor, HuCB 1957.2 and HuCB 1957.3; panel A), B31-A3Δ*vls* (ΔVlsE; pooled from 3 mice, donor HuCB 1957.2; panel B), or treated with lipopolysaccharide (LPS) and infected with ΔVlsE (pooled from 3 mice, donor HuCB 1957.2; panel C) were probed against whole-cell lysates of B31 (~10^7 cells per lane). Sera pooled from 2 uninfected mice (donor HuCB 1957.3) served as a negative control (panel D). A human whole blood sample received from the Lyme Disease Biobank served as a positive control for both anti-borrelia IgM and IgG antibodies (panel E).

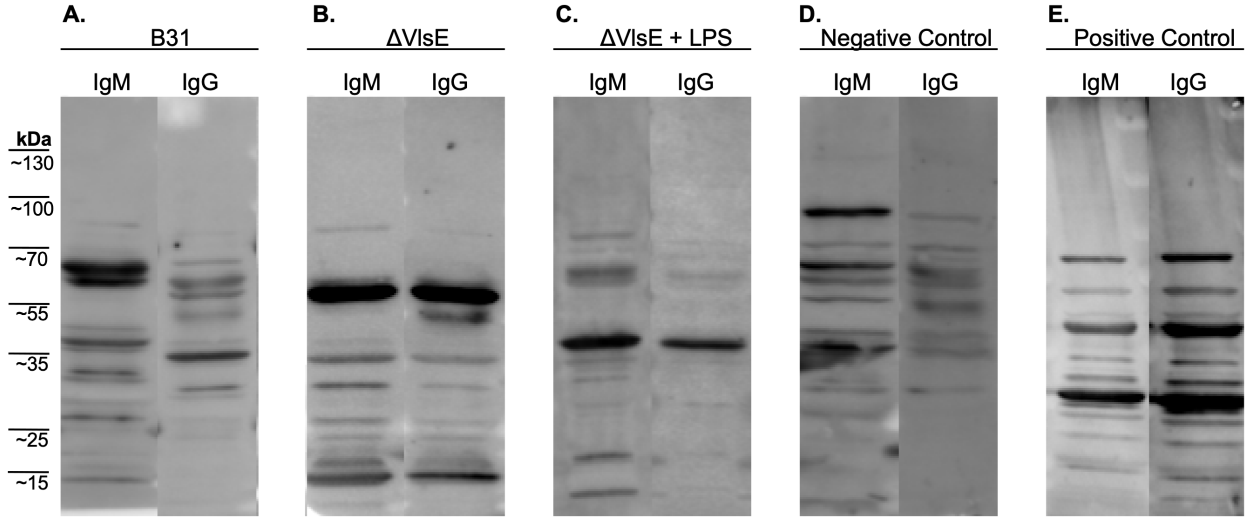

Figure S3. Immunoblots of sera pooled from huNOG-EXL SA mice and probed against whole-cell lysates of *Borreliella burgdorferi*. Pooled sera from huNOG-EXL SA mice (collected at day 41 postchallenge from donors 55 and 56) infected with *B. burgdorferi* B31-A3 (B31; pooled from 4 mice, donors 55 and 56; panel A), B31-A3Δ*vls* (ΔVlsE; pooled from 3 mice, donor 56; panel B), or treated with lipopolysaccharide (LPS) and infected with ΔVlsE (serum from 1 mouse, donor 56; panel C) were probed against whole-cell lysates of B31 (~10^7 cells per lane). Sera pooled from 3 uninfected mice (donor 56) served as a negative control (panel D). A human whole blood sample received from the Lyme Disease Biobank served as a positive control for both anti-borrelia IgM and IgG antibodies (panel E).

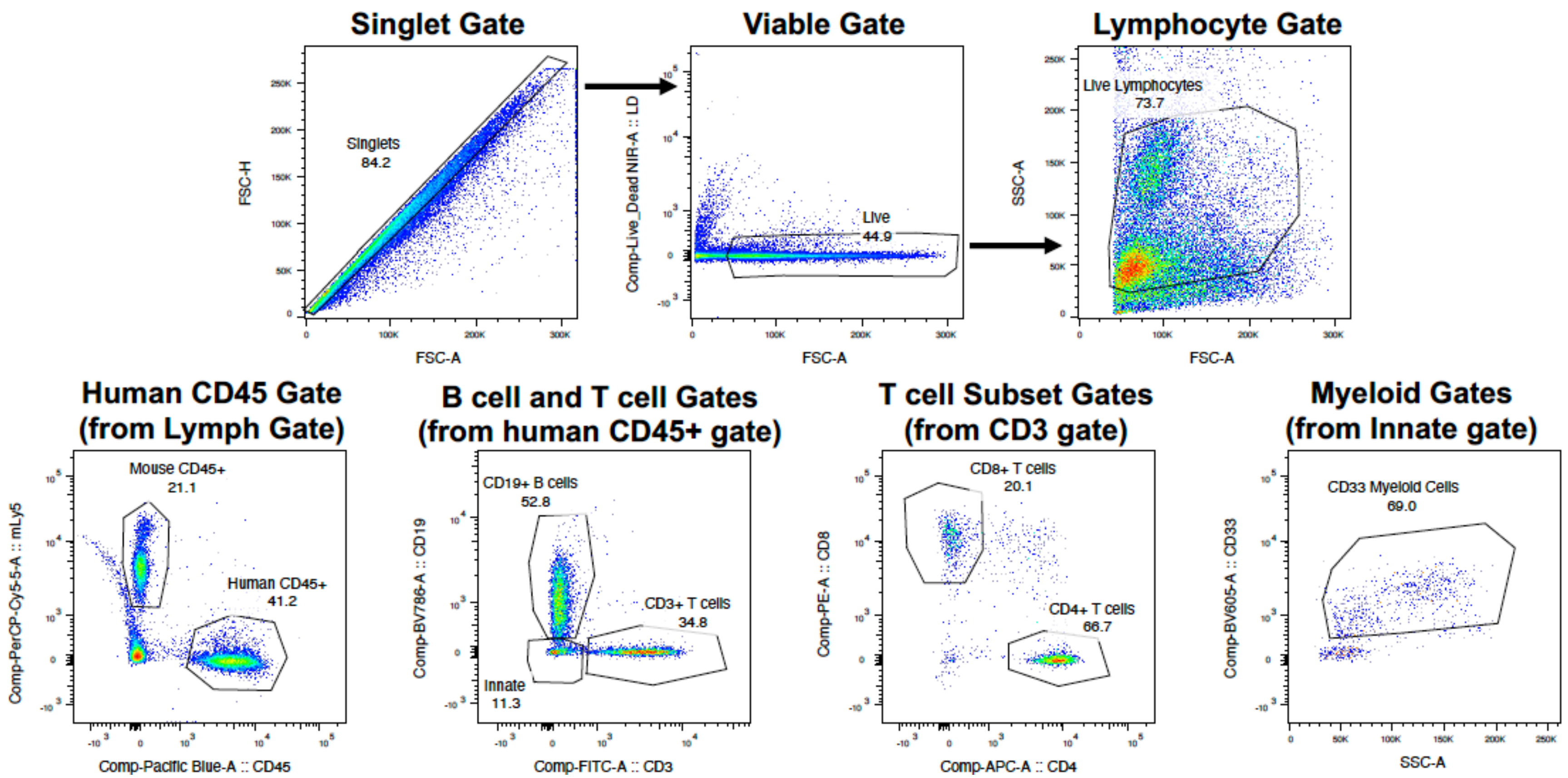

Figure S4. The representative flow cytometry data showing gating strategy.
